# Optimization of Spray Drying Process Parameters for the Preparation of Inhalable Mannitol-Based Microparticles Using a Box-Behnken Experimental Design

**DOI:** 10.3390/pharmaceutics15020496

**Published:** 2023-02-02

**Authors:** Jakub Karas, Sylvie Pavloková, Hana Hořavová, Jan Gajdziok

**Affiliations:** Department of Pharmaceutical Technology, Faculty of Pharmacy, Masaryk University, Palackého třída 1946/1, 612 00 Brno, Czech Republic

**Keywords:** large porous particles, spray drying, mannitol, inhalation, microparticles’ properties, process parameters, Box–Behnken design, multiple linear regression

## Abstract

Inhalation is used for local therapy of the lungs and as an alternative route for systemic drug delivery. Modern powder inhalation systems try to target the required site of action/absorption in the respiratory tract. Large porous particles (LPPs) with a size >5 μm and a low mass density (usually measured as bulk or tapped) of <0.4 g/cm^3^ can avoid protective lung mechanisms. Their suitable aerodynamic properties make them perspective formulations for deep lung deposition. This experiment studied the effect of spray-drying process parameters on LPP properties. An experimental design of twelve experiments with a central point was realized using the Box–Behnken method. Three process parameters (drying temperature, pump speed, and air speed) were combined on three levels. Particles were formed from a D-mannitol solution, representing a perspective material for lung microparticles. The microparticles were characterized in terms of physical size (laser diffraction), aerodynamic diameter (aerodynamic particle sizer), morphology (SEM), and densities. The novelty and main goal of this research were to describe how the complex parameters of the spray-drying process affect the properties of mannitol LPPs. New findings can provide valuable data to other researchers, leading to the easy tuning of the properties of spray-dried particles by changing the process setup.

## 1. Introduction

Pulmonary administration mediated by inhalation is an important non-invasive route of drug delivery [1,2]. This administration brings the possibility of both local and systemic effects using a wide range of different drugs [3,4]—from small molecules (beta-agonists or corticosteroids [5,6]) to large proteins (insulin [7]) or nucleic acids [1]. The lungs represent a target organ with several advantages: direct drug delivery to the site of action, bypassing the liver’s first-pass metabolism, large absorption surface, rich blood supply, reduced enzymatic activity, rapid onset of action, high bioavailability, and lower effective dose needed, etc. [1,8,9]. Delivery of the drug in the form of dry powder is more beneficial than liquid formulations. Dry powder inhalers are propellant-free, low-cost, portable, and easy to operate (no need for hand-and-breath coordination), and the drug’s dry state improves the formulation’s stability [2,10,11]. Dry particle usage brings more versatility of their properties control via particle engineering (particle size, surface morphology, mass density, stability, aerodynamic properties, charge, etc.) [12,13,14].

The particles have to possess the required properties for lung deposition. Their physical and, more importantly, aerodynamic diameter (mass median aerodynamic diameter—MMAD), are crucial among the others. MMAD is characterized as the diameter of a spherical particle of unit density with the same settling velocity as the particle of interest, regardless of its shape or density. Particles of MMAD larger than 10 μm do not reach the lungs and tend to deposit in the mouth and throat and are swallowed. Particles with MMAD 5–10 μm deposit dominantly in the oropharyngeal area and upper conducting airways but could be eliminated by mucociliary clearance. Particles 1–5 μm in diameter reach the deep lungs, represented by small airways and alveoli. Particles smaller than 0.5 μm are predominantly deposited by diffusion and easily exhaled [1]. 

The main disadvantage of dry powders containing particles small enough for deep lung deposition is their cohesiveness and poor flow properties. With decreasing geometric size, the inter-particulate van der Waals forces increase, leading to lower dispersion efficiency. Furthermore, even when deposited, these particles can be removed by macrophage activity before the drug can act or be available for absorption. A promising way to overcome these drawbacks is by using large porous particles [15]. LPPs have a much lower density than the conventional ones (<0.4 g/cm^3^), and thus it is possible to reach desired aerodynamic parameters with a physical size greater than 10 μm [12]. That improves the flowing properties and aerosolization of the powder from the device and protects the inhaled particles from macrophage clearance [15]. One approved product containing LPPs is tobramycin inhalation powder, represented by spherical sponge-like particles manufactured by PulmoSpheres^TM^ technology—spray drying of an o/w emulsion [16]. 

There are several methods for the preparation of dry powders for pulmonary delivery. Ordinarily, particle production is achieved by crystallization followed by micronization (milling) of a carrier material and API, subsequently blended. Usually, the coarse carrier crystals act as a host for micronized drug particles that are adhered to their surface (interactive mixtures) [17,18]. For instance, mixtures of salmeterol xinafoate and lactose were studied by Islam et al., concluding that adding very fine carrier particles to the formulation is beneficial for better performance [19]. Crystallization and milling procedures are burdened with disadvantages, such as inadequate control of particle size, surface properties, morphology, and electrostatic charge generation, which cause agglomeration, rapidly reducing aerosolization and, thus, bioavailability [20,21]. Novel methods such as freeze drying, spray drying, spray freeze drying, or supercritical fluid technologies bring better control over these parameters [22,23]. Freeze drying (lyophilization) of mannitol solutions showed that this technique provided poor control over the resulting particles as they had a wide size distribution and bad flow properties. However, freeze-dried mannitol generated weak adhesive forces with salbutamol sulfate thanks to a smoother surface [24]. High porosity and volume are typical for spray-freeze-dried particles, but only a limited amount of API can be loaded [4]. For example, van Drooge et al. successfully prepared inhalable particles containing tetrahydrocannabinol in an inulin carrier [25]. In supercritical assisted spray drying, a mixture of supercritical CO_2_ and feed dispersion is sprayed into the heated drying chamber. The CO_2_ expands from the droplets and creates secondary smaller droplets. This process is ecological and suitable for (thermo)sensitive materials such as proteins [26]. For example, Du et al. incorporated lysozyme into particles of suitable size with no impact on its secondary structure [27]. 

Among these methods, spray drying is still preferred due to the possible control of the final particle properties and cost-effectiveness. It is a fast, one-step method with an optimal yield and high reproducibility [28]. Several variables play an important role in influencing the resulting particle properties. Higher atomization pressure leads to the formation of smaller droplets. Reduction in droplet size is also achieved by downregulating the pump speed rate. The opposite effect is caused by increasing the viscosity of the input dispersion or its high surface tension. Inlet temperature has a major impact on final moisture content and drying rate and, thus, particle shape and morphology. Finally, air speed rate influences the effectiveness of drying and particle separation [28,29,30,31]. 

The selected carrier material greatly impacts the particle properties and their behavior in the respiratory tract. The most widely used material is lactose. Lactose is produced from bovine milk, which raises concerns about getting transmissible spongiform encephalopathy (TSE) disease. Lactose products are not optimal for patients with lactose intolerance or diabetes mellitus. Moreover, it may be incompatible with specific APIs (e.g., formoterol or proteins) as it interacts with their functional groups due to its reducing sugar function. For these reasons, the pharmaceutical industry is looking for alternative materials for dry powder formulation. Different carbohydrates (trehalose, raffinose), polyols (mannitol, sorbitol, erythritol), or polymers (chitosan, PLGA) have been investigated [10]. For this experimental work, mannitol was chosen for its non-animal origin and lower hygroscopicity than lactose. It is a non-reducing polyalcohol with a sweet taste, which could indicate proper dosing. It is also approved as a GRAS substance by the FDA and the European Regulatory Committee [32]. It is currently marketed for diagnostic (Aridol^TM^) and therapeutic (Bronchitol^TM^) purposes as a dry powder inhaler [10]. 

Nowadays, the optimization of process/formulation parameters represents an important step in research, and the design of experiment (DoE) approach is getting great attention in pharmaceutical technology [33]. Response surface methodology (RSM) is widely used to understand the relationship between input variables and response(s) or to find optimal process settings while simultaneously achieving high efficiency and low costs. A suitable tool for RSM application when having 2–4 independent factors is the Box–Behnken design (BBD) [34], which requires fewer factor combinations than a central composite design and is rotatable or nearly rotatable, still sufficient to fit a quadratic model. BBD is also beneficial when avoiding the extreme factor level combinations is desirable.

The influence of process parameters on mannitol particles properties was investigated using different DoE approaches and statistical methods. For example, Littringer et al. used a full-factorial design and stated that higher temperatures lead to a smoother surface and lower feed concentration. High atomizer rotation speeds created smaller particles [35]. Kramek-Ramanowska et al. concluded that the minimum particle size is mainly determined by a low drying temperature and a high gas flow rate through the nozzle using the same DoE type [36]. According to Li et al., aerosol performance is better with increasing pump speed rate based on DoE analysis [37]. With the application of BBD, Guimarães et al. showed the effect of outlet temperature and feed concentration on particle size and shape [38].

The present study investigated the influences of three less-discussed process parameters of spray drying (drying temperature, pump speed, and air speed) on the various responses (properties of LPPs, especially particle size characteristics). A BBD and analysis of DoE data were employed to provide the best mathematical models of the mentioned dependencies using multiple linear regression (MLR) and to visualize results via perspective plots. This study’s main goals and novelty were to describe the influences of studied process variables on mannitol LPP features and to provide usable new findings to other research groups trying to tune the properties of spray-dried particles by changing the process setup.

## 2. Materials and Methods

### 2.1. Spray Drying Process

A LabPlant spray dryer (SD-06, LabPlant, Filey, UK) with a drying chamber (215 mm in diameter and 500 mm long) was used for particle formulation. A spray solution of 10% (*w*/*w*) D-mannitol (Penta, CZ; Mr = 182.18) in distilled water was prepared in laboratory conditions at temperatures up to 25 °C. For atomization, a two-fluid, 2 mm nozzle was used. The drying conditions for the experiment were combined into three levels (Table 1). The inlet temperature of the drying gas was set to 100 °C, 120 °C, or 140 °C, respectively. The feed rate of the primary solution was determined by pump speeds of 5 mL/min, 10 mL/min, and 15 mL/min, respectively. The last process parameter air speed was 3.0 m/s, 3.6 m/s, and 4.2 m/s. Constant atomizing pressure was set to 300 kPa. The final product was separated through a cyclone and stored in glass bottles in an exicator for a maximum of 10 days before evaluation.

### 2.2. Particle Size and Aerodynamic Diameter

Each sample was dispersed in 96% ethanol. The suspension was analyzed by laser diffraction (LA-960, Horiba, Kyoto, Japan). Before measuring, a small amount (<1 g) of each sample was dispersed in ethanol, sonicated for 30 s, and stirred for maximal homogeneity of the suspension. Refractive index settings were: ethanol RI = 1.360, mannitol RI = 1.333, and imaginary index iRI = 0, respectively. Measurement was performed according to the recommended settings [39]. Each measurement of the laser diffraction median size (S, μm) was performed in three repetitions and presented as a mean with geometric standard deviation. 

Aerodynamic properties were measured by an APS (aerodynamic particle sizer—TSITM model 3321, TSI Incorporated, St. Paul, MN, USA). The method is based on measuring the time of flight of a particle over a well-defined distance between two lasers with detectors. Firstly, the sample is dispersed by the compressed air using the TSI small-scale powder disperser 3433 USA (set parameters: air speed = 5 L/min, air pressure = 200 kPa). Then, the sample goes through the nozzle, where the flow of the particles is accelerated. The velocity of the particles is measured in time in the detection area. Measurement was performed according to the recommended settings [40]. Mass median aerodynamic diameter (MMAD, μm) and numeric median aerodynamic diameter (NMAD, μm) were detected. Each measurement was performed in three repetitions and presented as a mean with geometric standard deviation. 

### 2.3. Bulk and Tapped Density, Hausner Ratio

For density measurements, 5 mL of each sample was used. The bulk density was calculated from the weight and volume according to the European Pharmacopeia. Tapped density was measured by Erweka^®^ model SVM102 (DE). The volumes of samples were reported after 10, 500, 1250, and 2500 taps. The Hausner ratio was calculated using tapped (after 2500 taps) and bulk density values [41]. 

### 2.4. Particle Morphology

The morphology and surface structure of mannitol LPPs were characterized by scanning electron microscopy (SEM). Samples were placed onto aluminum stubs with double-sided adhesive carbon tape, coated with a 10 nm gold layer (sputter Q150R, Quantum Technologies, London, UK), and visualized using an SEM (MIRA3, Tescan, Brno, Czechia). Obtained signals of the samples were produced by secondary electrons (SE) at 5 kV voltage and 5 kx magnification. Particles were classified on a three-point scale as G—good, A—acceptable, and B—bad (Table 2). The integrity of the surface, spherical shape, uniformity of size distribution, and presence of fragments were monitored. Spherical, integral, uniformly wrinkled, and size-uniform particles are marked as good—G. Oval, integral with damages, non-homogeneously wrinkled, or partially wrinkled particles with larger differences in size are marked as acceptable—A. Particles with an irregular shape, a high proportion of damaged particles, wide size distribution, and a high fragment content are marked as bad—B. To build a mathematical model, the morphology was expressed on the coded scale 1–3 (3 = G—good, 2 = A—acceptable, 1 = B—bad). Despite the discrete nature of the obtained response, the assumption is that the particle morphology grade for various process parameter settings within the DoE region can take on values approximately in the range of 1–3.

### 2.5. Experimental Design

The three-factorial and a three-level BBD with three replications of the center point were applied for DoE, resulting in 15 experimental runs to assess the effect of input parameters on various product quality attributes. The process parameters: drying temperature (X_1_; in the range of 100–140 °C), pump speed (X_2_; in the range of 5–15 mL/min), air speed (X_3_; in the range of 3.0–4.2 m/s) were selected as studied input variables. Each factor was divided into three levels coded as low (−1), medium (0), and high (+1), as listed in Table 1, which were chosen based on previous experiments with the production of LPPs used in experiments on an artificial lung model to simulate particle deposition during different breath cycles [42].

Utilized BBD is a cubic design, where the 12 experimental runs are represented by the mid-point of each edge of the 3D cube, and the 13th point is the center point coded as a set of factors: 0, 0, 0. The experimental runs were carried out randomly to minimize the effect of unexplained variability on observed responses. Replications of the center point were added to ensure process stability and check the model curvature. They were dispersed as evenly as possible throughout the BBD matrix (the 1st, the 8th, and the 15th experimental run). The measured characteristics of microparticles in the present study were S (Y_1_, μm), MMAD (Y_2_, μm), NMAD (Y_3_, μm), relative standard deviation (RSD) of MMAD (Y_4_, %), particle morphology characterized by SEM (Y_5_; in the coded scale 1–3; higher grade is better), and flow properties (bulk density, tapped density, and Hausner ratio).

### 2.6. Data Analysis

DoE analysis in this study included the following steps (generalized procedure):

1. Data checking; descriptive statistics; basic data visualization (histograms, box plots).
2. Choosing a suitable mathematical model for the obtained data.
3. Testing the assumptions required for ANOVA and regression analysis (MLR), primarily using visual assessment of graphical outputs (histograms, residual graphs); eventually, exclusion of the outliers from the analysis and subsequent building of a new model.
4. Simplification of the model by gradual backward elimination of insignificant terms (assessment of their *p*-values in ANOVA table) while monitoring and comparing values of coefficient of determination R^2^, adjusted R^2^, Akaike information criterion, and *p*-value of the original and simplified model to achieve the best quality of the model fit. Percentage prediction error (PPE) was assessed to ensure the validity of the generated regression equation. PPE for each experimental run was calculated as: (observed value—predicted value)/predicted value × 100 (%).
5. Visualization of MLR results: RSM via 3D perspective plots.
6. Interpretation of obtained regression equation and graphical outputs: investigation of the effects of independent factors (process conditions) and their possible interactions on the response (quality attributes of microparticles) and the determination of optimal process conditions for the desired response.

The results of MLR were fitted to a quadratic polynomial model given by equation (Equation (1)):

(1) $$Y={\;b}_{0}+b_{1}X_{1}+{\;b}_{2}X_{2}+b_{3}X_{3}+b_{12}X_{1}X_{2}+b_{13}X_{1}X_{3}+b_{23}X_{2}X_{3}+b_{11}X_{1}^{2}+{\;b}_{22}X_{2}^{2}+b_{33}X_{3}^{2}$$

where Y stands for the estimated response; X_1_, X_2_, and X_3_ are independent variables; b_0_ is an intercept/constant; b_1_, b_2_, and b_3_ are regression coefficients for linear terms; b_12_, b_13_, and b_23_ are regression coefficients for interaction/cross product terms; b_11_, b_22_ and b_33_ are regression coefficients for quadratic terms. Standardized regression coefficients (β) were also determined to compare the influence of individual terms on the response. 

Data analysis was performed with a significance level of α = 0.05, so the effects at *p* < 0.05 were considered statistically significant. The design of the experiment and subsequent analysis of the obtained data were carried out in R software, version 4.2.1 [43]. 

## 3. Results and Discussion

All results obtained are presented in Table 2. Measured were: laser diffraction median size value (Y_1_; S, μm) and mass median aerodynamic diameter (Y_2_; MMAD, μm) as the most pronounced parameters in the research. Numerical median aerodynamic diameter (Y_3_; NMAD, μm) provides important information about the numerical distribution of particles. Y_4_ represents the RSD of MMAD. Finally, scanning electron microscopy was performed as an important indicator of surface and morphological properties (Y_5_; SEM, values: G—good, A—acceptable, B—bad). Other measured values are bulk density (g/cm^3^), tapped density (g/cm^3^), and Hausner ratio.

To assess the combined effects of three factors (drying temperature, pump speed, and air speed) on the various responses (particle size parameters and morphology), a BBD of 15 experimental runs in conjunction with subsequent data analysis was used. By performing multiple regression analysis on the experimental data, a second-order polynomial equation for each response variable was obtained (expressed in tabular arrangement). The resulting MLR models, including regression coefficients for each term, corresponding *p*-values, and selected model characteristics (R^2^, adjusted R^2^, *p*-value), are summarized in Table 3. The mean values of each response variable for each experimental run, as well as the values predicted by MLR models (from Table 3) and errors of prediction, are reported in Table 4. This evaluation is presented for quantities for which a significant influence of the process parameters on the final value was revealed using statistical testing. The raw data, with indicated outliers excluded from the subsequent analysis, are presented in Table S1.

### 3.1. Laser Diffraction Median Size—S (Y_1_)

**Figure 1:**
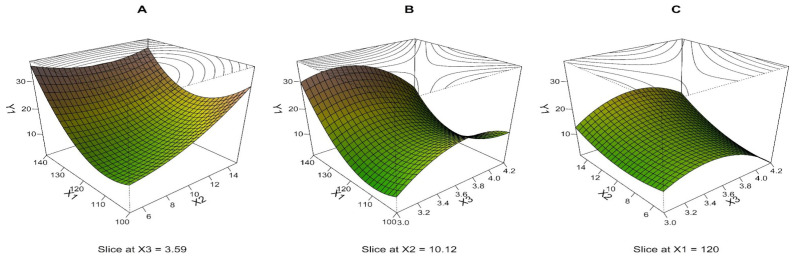
Three-dimensional (3D) perspective plots: response surface of S (Y_1_, μm) as a function of drying temperature (X_1_, °C), pump speed (X_2_, mL/min), and air speed (X_3_, m/s).

The MLR model of S was built using 15 experimental runs with three repeated measurements. Three outliers were excluded from the analysis based on the residual plots. The quadratic model, including all terms, was chosen as the best-fitting model. The R^2^ value of 0.813 means that the model can clarify 81.3% of the variability, and the associated *p*-value of less than 0.001 indicates the model is highly significant (Table 3). PPE for most runs is in the range of several units up to tens of percent, except for run 11—in this DoE region, the predictive ability of the model sharply decreases (extremely high PPE value, 748.9%). The mean PPE value is relatively high (67.6%). If run 11 is not considered, the mean PPE value is 18.9%, which is acceptable. Based on the mentioned characteristics, the model fitting can be considered satisfactory for response determination, especially in identifying independent variables’ effects on the S value. Based on the standardized regression coefficient values (Table 3, β values), drying temperature and air speed (linear and quadratic terms in both cases) have the most considerable effect on the response. Pump speed and interactions between variables also contribute to the model (Figure 1).

The interrelationships between process parameters and S, closely defined by the quadratic equation, are depicted via perspective plots, where the effects of two input variables and their interaction at the middle level of the third variable are visualized. The middle level of the third input variable is marked as “slice at” in the graph, and it is calculated as the mean for all measurements after excluding outliers; therefore, it may not exactly correspond to the medium level (0) from Table 1. It is further described, like other monitored microparticle properties, in Section 3. It could be seen that there was a considerable variation in the S value with different process parameter settings (Figure 1). The perspective plot in Figure 1A shows that S rapidly increased up to the values above 30 μm with increasing drying temperature (X_1_) or pump speed (X_2_), as well as when combining the high values of these variables.

This observation follows the Broadhead et al. study, which suggested that the material’s rapid agglomeration can cause the formation of larger particles at higher temperatures [44]. In the study published by Stahl et al., it was observed that increasing temperature significantly influences physical particle size. High inlet temperature leads to the formation of the largest particles. Another finding of this study was that particle size decreased when air speed increased. This effect is caused because higher air speed produces more energy for aerosolization (conversion of the primary dispersion into the droplets), resulting in smaller droplets [45]. This finding was confirmed partly in our study due to the particles’ physical size affection by other process parameters (not set on the same level in the compared study). 

As shown in Figure 1, using the low to medium values of X_1_ and X_2_ seems to be the most appropriate for achieving particles of around 10 μm. The saddle shape of the response surface in Figure 1B,C suggests a more complex dependency, where low or conversely high air speed (X_3_) in combination with low drying temperature (X_1_) or low pump speed (X_2_) appears to be the experimental settings leading to the S less than 10 μm. The experimental region around the center point results in a response slightly above 10 μm and thus represents the optimal conditions for preparing LPPs suitable for lung deposition.

From the measured sample set, microparticles with the optimal S value are provided by central point—experimental run 13 (mean and SD for 13a, 13b and 13c − S: 13.34 ± 4.26 μm, X_1_: 0, X_2_: 0, X_3_: 0), 7 (S: 11.08 ± 3.65 μm, X_1_: −1, X_2_: 0, X_3_: +1), 9 (S: 12.75 ± 4.03 μm, X_1_: 0, X_2_: −1, X_3_: −1), and 12 (S: 11.08 ± 4.10 μm, X_1_: 0, X_2_: +1, X_3_: +1) [12]. 

### 3.2. Mass Median Aerodynamic Diameter—MMAD (Y_2_)

BBD, including 15 experimental runs with 3 repeated measurements, was used to obtain an MLR model of MMAD. Based on the residual plots, three outliers were excluded from the data analysis. MLR proposes the quadratic model, including all terms, as the best-fitting model. The R^2^ value, which is found to be 0.810, suggests that the model can explain 81.0% of the total variability. The detected *p*-value of less than 0.001 implies that the regression equation is significant (Table 3). The low mean PPE (1.9%) indicates the high predictive ability of the MLR model. Considering these facts, it can be concluded that the resulting model is adequate for MMAD response prediction under various experimental conditions within the range of the analyzed BBD. It can be seen from Table 3 that the importance of the effect of individual process parameters on MMAD decreases in the following order: drying temperature > air speed > pump speed (applies to both linear and quadratic terms of the mentioned variables). 

**Figure 2:**
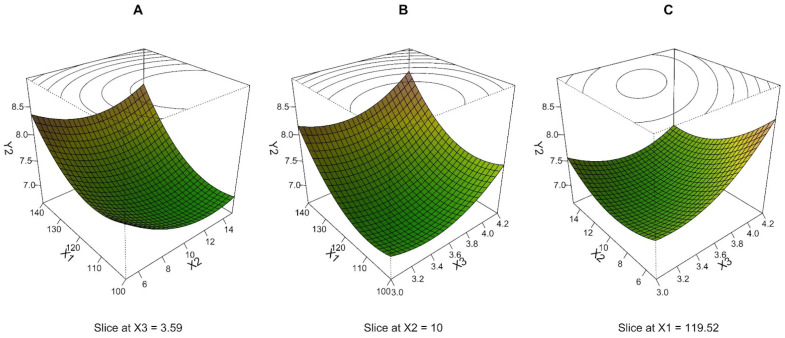
Three-dimensional (3D) perspective plots: response surface of MMAD (Y_2_, μm) as a function of drying temperature (X_1_, °C), pump speed (X_2_, mL/min), and air speed (X_3_, m/s).

The interrelationships between the dependent variable and independent parameters were further elucidated using perspective plots. Response surface as the combination of two process parameters by considering the third parameter at a middle level is presented in Figure 2. MMAD dropped to a value around 7 μm when the drying temperature (X_1_) and air speed (X_3_) decreased and pump speed (X_2_) increased, while under other conditions within the experimental design it reached values up to 8.2–8.4 μm. Especially high X_1_ values (Figure 2A,B) and a combination of X_2_ low level and X_3_ high level (Figure 2C) resulted in a quite high MMAD value. On the other hand, the center point and the region around (0, 0, 0) correspond to the appropriate process conditions leading to the small particles.

Experimentally, the MMAD was lowest in experimental runs: 13 (mean of samples 13a,13b,13c MMAD: 7.02 ± 2.75 μm, X_1_: 0, X_2_: 0, X_3_: 0), 3 (MMAD: 7.07 ± 2.71 μm, X_1_: −1, X_2_: +1, X_3_: 0), 9 (MMAD: 7.19 ± 6.68 μm, X_1_: 0, X_2_: −1, X_3_: −1), and 5 (MMAD: 7.20 ± 2.62 μm, X_1_: −1, X_2_: 0, X_3_: −1) (Table 2). Values were achieved at medium and low process parameter values for air speed and drying temperature, while pump speed was at a middle and high level, except in run 9, where the pump speed was at level −1.

Maltesen et al. used a similar experimental setup in their study aimed at inhalation insulin particle preparation. Process parameters (drying temperature, pump speed, and air speed) were set at three levels. Like our experiment, only small differences in aerodynamic diameters between samples were observed, a range of 2.57–3.71 μm (compared against one level of insulin concentration. It was selected according to the largest number of samples for a more accurate comparison). However, the lowest values were also achieved with medium settings of the process parameters [46].

### 3.3. Numeric Median Aerodynamic Diameter—NMAD (Y_3_)

For NMAD, the MLR model using 15 experimental runs of BBD with 3 repetitions was calculated. The exclusion of two outlier measurements was based on the residual plot indication. The MLR method provided a prediction equation including a quadratic term only for pump speed as a statistically significant coefficient; the quadratic effect of other variables (drying temperature and air speed) and their mutual interaction have not been assessed as significant by ANOVA. The quality of the resulting model was evaluated by R^2^ value, *p*-value, and PPE. The coefficient of determination (0.679) does not indicate a very strong goodness of fit. In contrast, the mean value of PPE is low enough (4.2%), which, on the contrary, indicates a good prediction ability (Table 3). The overall model *p*-value of less than 0.001 demonstrates that the regression equation is significant. Therefore, the resulting model can still be used to determine the dependencies occurring in the data matrix. Based on the regression coefficient values listed in Table 3, it can be deduced that the quadratic term of the pump speed represents the greatest influence on the NMAD. The interaction terms and linear terms of drying temperature and air speed have a lower effect on the response, but they are still significant. 

**Figure 3:**
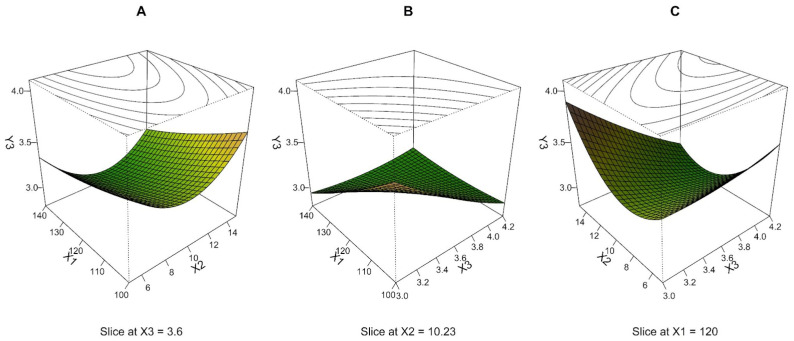
Three-dimensional (3D) perspective plots: response surface of NMAD (Y_3_, μm) as a function of drying temperature (X_1_, °C), pump speed (X_2_, ml/min), and air speed (X_3_, m/s).

Response surfaces in Figure 3 were plotted based on the quadratic model, with all terms as the combination of two independent variables at a middle level of the third one. The shape of the response surface in Figure 3A indicates that NMAD decreases with increasing drying temperature (X_1_) at any value of pump speed (X_2_). Still, this effect is most pronounced at X_2_ medium level. A similar dependence can be observed in Figure 3C, where there was a descending response with the increment in air speed (X_3_) at various X_2_. Still, again, the steepest descent manifested at X_2_ medium level. As shown in Figure 3B, a visual analysis of the depicted response surface plot indicates that the NMAD decreases as the X_1_ and X_3_ increase. For all response surfaces, the NMAD values decreased to approximately 3 μm while the upper parts of the response surface were close to 4 μm. Although the dependences determined by MLR, in this case, differ from the response surfaces for the variable Y_2_ (compare Figure 2 and Figure 3), the center point and the region around (0, 0, 0) can again be evaluated as a suitable combination of process parameters resulting in an optimal response.

The experimental NMAD results ranged from 2.68 to 4.14 μm (Table 2), which can be considered satisfying. However, RSDs of NMAD reached enormous values (over 100%) in most cases, making these results inaccurate. However, the spray drying method generates polydisperse particles, which correspond to higher values of NMAD RSD [47].

### 3.4. Relative Standard Deviation of Mass Median Aerodynamic Diameter—MMAD RSD (Y_4_)

**Figure 4:**
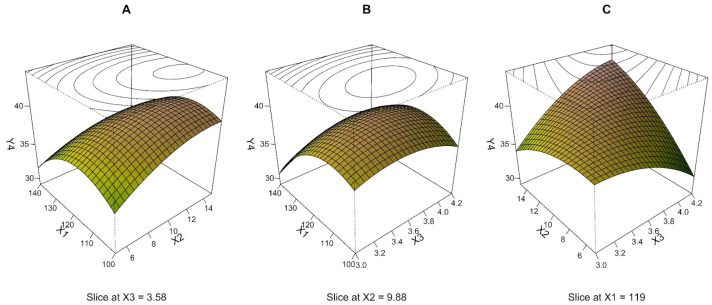
Three-dimensional (3D) perspective plots: response surface of MMAD RSD (Y_4_, %) as a function of drying temperature (X_1_, °C), pump speed (X_2_, ml/min), and air speed (X_3_, m/s).

An MLR model of MMAD RSD was built on BBD with 15 experimental runs and 3 repeated measurements. Based on the residual plots, two outliers were excluded from the data analysis, and another three measurements that were excluded during MMAD model development were not included in the calculations either. A quadratic model including all terms was generated and assessed as the most accurate and meaningful model. The R^2^ value of 0.775 implies that 77.5% of the variability can be interpreted by the model, which is acceptable. The statistical significance of the model was confirmed by the *p*-value (*p* < 0.001) (Table 3). Predicted errors of all runs within 5% (the mean value of 2.2%) demonstrate the excellent fit of the MLR model. All the results mentioned above clearly show that the quality of the model is satisfactory and reliable, especially for examining data dependencies in the DoE region. As presented in Table 3, the linear and quadratic coefficients of drying temperature were the terms that most affected the response. Other quadratic terms and interactions between pump speed and air speed contribute less to the model but are still statistically significant, as can also be inferred from Figure 4.

Since the regression model had three independent variables, one variable was fixed at a constant at a middle level for each perspective plot (Figure 4). Values of MMAD RSD range in the tens of percent and increase to a maximum of about 40%, especially in the area around the central point, as can be seen from the shape of the response surface in Figure 4A,B. With a gradual increase in drying temperature (X_1_) at any pump speed (X_2_) or any air speed (X_3_), a decrease in MMAD RSD to values approaching 30% can be observed. From Figure 4C, an interaction between X_2_ and X_3_ can be deduced. High values of MMAD RSD are achieved, especially at high levels of both independent variables. A decrease in MMAD RSD can be obtained with a combination of experimental conditions: high X_2_ at the low level of X_3_ or, conversely, low X_2_ at the high level of X_3_. Therefore, the region around the central point (0, 0, 0) appears less suitable for particle size variability. 

Experimentally, MMAD RSD is in the range of 30.0–42.5%, representing corresponding values for the spray drying method. Spray drying, like most methods, leads to the formation of polydisperse particles with a variety of distributions in their sizes. This parameter is, therefore, important just in the overall spectrum of evaluation. The most pronounced parameters for LPPs are laser diffraction median size, mass median aerodynamic size, and particle morphology [48].

### 3.5. Particle Morphology Characterized by SEM (Y_5_)

For particle morphology, an MLR model based on 15 experimental runs of BBD was established. In terms of quadratic coefficients, the MLR method provided a prediction equation that included a statistically significant quadratic term for only air speed. In contrast, the quadratic effect of pump speed and drying temperature was insignificant according to the ANOVA table. Model competency was validated using the following parameters: R^2^ value, *p*-value, and PPE. The value of the determination coefficient (0.866) shows that the model fitting is relatively good. The mean PPE (13.7%) indicates acceptable magnitudes of differences between experimental and calculated data (Table 3). The results may also be affected by the relatively low number of experimental values (absence of repeated measurements for each run), given the nature of the discussed microparticles’ properties. The model *p*-value of 0.012 proves the statistical significance of the identified dependencies. Therefore, the found equation can be used to display the regression equation and to explain these relationships. From Table 3, it can be seen that the air speed has a considerable influence on the response. The lower magnitude of other terms indicates their lower effects; however, some are still statistically significant. 

**Figure 5:**
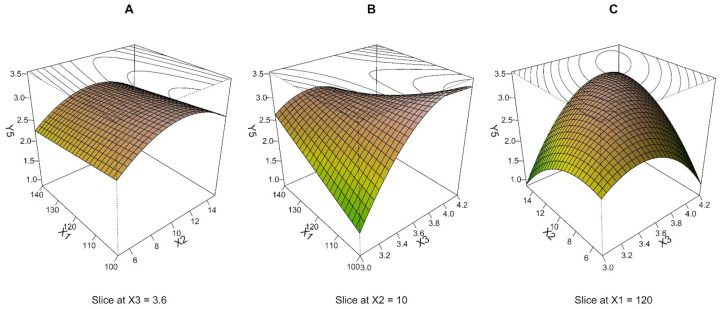
Three-dimensional (3D) perspective plots: response surface of particle morphology (Y_5_, the scale 1–3, the higher grade is better) as a function of drying temperature (X_1_, °C), pump speed (X_2_, mL/min), and air speed (X_3_, m/s).

Figure 5 displays the surface profiles as the response of varying values of two independent factors at a fixed level (0) of the third one, whereas the quadratic model with all terms was considered. Better particle morphology can be achieved at the middle level of pump speed (X_2_) and air speed (X_3_), irrespective of the drying temperature (X_1_), as can be seen in Figure 5A,B. From Figure 5C, it can be interpreted that the combination of medium-to-high levels of X_2_ and X_3_ leads to the maximum particle morphology grade, shown in the plot as a peak value of 3. Thus, it can be concluded that the experimental conditions corresponding to the region around the center point (0, 0, 0) result in the microparticles with the best particle morphology.

**Figure 6:**
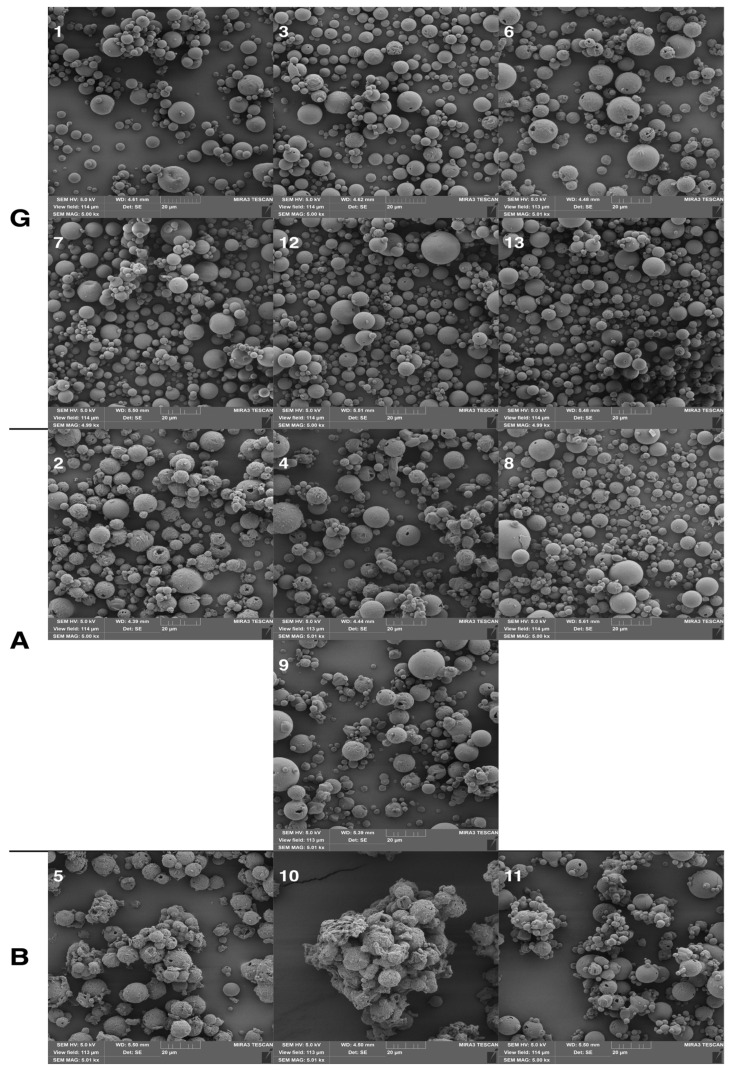
SEM images of samples divided into 3 categories: G—good; A—acceptable; B—bad (magnification 5k×).

The experimental finding showed that samples 7 (X_1_: −1, X_2_: 0, X_3_: +1), 9 (X_1_:0, X_2_: −1, X_3_: −1), 12 (X_1_: 0, X_2_: +1, X_3_: 01), 13 a, b, c (X_1_: 0, X_2_: 0, X_3_: 0) have process parameters set to provide particle sizes S (parameter Y_1_, μm) in the range 11.08 ± 3.65 μm–13.34 ± 4.26 μm, which is the closest to ten targeted micrometers. Particle morphology provided by SEM pointed out spherical, polydisperse particles with minimal damage. These particles are marked as good (G) except for sample 9. In Figure 6, sample 9 (X_1_: 0, X_2_: −1, X_3_: −1) spherical particles with a larger size distribution, locally wrinkled with few holes present, and very few fragments can be seen. Similar observations were observed in samples 2 (X_1_: +1, X_2_: −1, X_3_: 0), 4 (X_1_: +1, X_2_: +1, X_3_: 0), and 8 (X_1_: +1, X_2_: 0, X_3_: +1). These particles are marked as acceptable—A. Spherical polydisperse particles, with a small presence of holes, with suitable shapes were observed in samples 1 (X_1_: −1, X_2_: −1, X_3_: 0), 3 (X_1_: −1, X_2_: +1, X_3_: 0), 6 (X_1_: +1, X_2_: 0, X_3_: −1), 7 (X_1_: −1, X_2_: 0, X_3_: +1),12 (X_1_: 0, X_2_: −1, X_3_: +1), and 13 a, b, c (X_1_: 0, X_2_: 0, X_3_: 0), these samples are classified as good (G). In samples 5, 10, and 11, particles with an irregular shape, a high proportion of damage, wide size distribution, a high fragment content, and a tendency to agglomerate can be observed. These are marked as bad (B).

The influence of drying temperature, pump speed, and air speed on bulk, tapped density, and Hausner ratio (and mutual interactions of process factors) have not been confirmed as statistically significant using ANOVA (all *p* > 0.05). 

All samples showed densities < 0.4 g/cm^3^, which fulfills the requirement for LPPs according to current knowledge (Table 2). Bulk density values ranged between 0.25 and 0.31 g/cm^3^ and tapped densities were between 0.34 and 0.41 g/cm^3^. These small differences are not considered statistically significant. The Hausner ratio values as the parameter of powder flow (important for processing powders into capsules for DPI filling) ranged from 1.25 to 1.47, which corresponds to acceptable to bad flow behavior [1,41,49]. 

In summary, as follows from the discussion of the individual responses and the significance of the regression model coefficients (Table 3), the crucial factor influencing the resulting particle properties is drying temperature. Increasing drying temperature leads to the formation of larger particles in physical diameter (S) and aerodynamic size (MMAD). Higher feed rate (pump speed) led to the formation of larger particles in physical diameter. Increasing the air speed tends to produce particles with a larger MMAD. 

In the promising samples, the MMAD was around 7 μm, which is still greater than the optimal range (1–5 μm). However, sample 13 (central point) represents particles with the lowest MMAD and a passable S. The central point appears to have an optimal setting of process parameters for expanding and incorporating other parameters (e.g., concentration and atomization pressure) or for fine-tuning the properties of the spray-dried LPPs to reach the deep lung region. 

## 4. Conclusions

The main purpose of this study was to determine the spray-drying process parameters affecting mainly the physical size (S) of the prepared LPPs and their aerodynamic properties (MMAD, NMAD). Other parameters, such as particle morphology, bulk, tapped density, and HR, were less important. The particles’ size and aerodynamic diameter were significantly affected by the variation of all three considered process parameters (drying temperature, pump speed, and air speed). In general, the effect of spray-drying process parameters on the properties of mannitol LPPs was described and evaluated. The region around the DoE central point (the drying temperature of 120 °C, the pump speed of 10 mL/min, and the air speed of 3.6 m/s) was found optimal for achieving the desired response of the monitored particles’ characteristics. The innovative presented data can provide other research groups with a description of how to tune spray-dried particles properties by changing the process setup. 

## Figures and Tables

**Table 1:** Independent variables and levels of BBD.

Input Variable (Process Parameter)	Unit	Code Level		
		Low (−1)	Medium (0)	Hight (+1)
Drying temperature (X_1_)	°C	100	120	140
Pump speed (X_2_)	mL/min	5	10	15
Air speed (X_3_)	m/s	3.0	3.6	4.2

**Table 2:** Results of experimental measurements.

Run	Drying Temperature (X_1_)	Pump Speed (X_2_)	Air Speed (X_3_)	S (Y_1_)	MMAD (Y_2_)	NMAD (Y_3_)	MMAD RSD (Y_4_)	SEM (Y_5_)	Bulk Density	Tapped Density	Hausner Ratio
	[°C]	[mL/min]	[m/s]	[μm]	[μm]	[μm]	[%]	[Grade]	[g/cm^3^]	[g/cm^3^]	
1	100	5	3.6	9.13 ± 3.40	7.67 ± 2.69	3.60 ± 3.24	35.2	G	0.31	0.39	1.28
2	140	5	3.6	26.33 ± 3.98	8.28 ± 2.67	3.65 ± 3.60	32.3	A	0.29	0.38	1.32
3	100	15	3.6	39.39 ± 4.81	7.07 ± 2.71	3.48 ± 3.19	38.4	G	0.25	0.34	1.39
4	140	15	3.6	35.80 ± 5.32	8.27 ± 2.68	3.22 ± 4.51	32.4	A	0.29	0.37	1.28
5	100	10	3.0	5.60 ± 3.45	7.20 ± 2.62	3.70 ± 3.59	36.4	B	0.30	0.41	1.37
6	140	10	3.0	32.07 ± 5.10	8.42 ± 2.89	2.68 ± 4.93	30.0	G	0.28	0.35	1.25
7	100	10	4.2	11.08 ± 3.65	7.34 ± 2.66	3.20 ± 1.14	36.3	G	0.27	0.39	1.43
8	140	10	4.2	18.34 ± 3.88	8.33 ± 2.87	3.02 ± 3.70	34.8	A	0.27	0.40	1.47
9	120	5	3.0	12.75 ± 4.03	7.19 ± 2.68	3.47 ± 3.43	37.2	A	0.25	0.35	1.39
10	120	15	3.0	8.22 ± 3.23	7.33 ± 2.58	4.14 ± 2.78	35.2	B	0.26	0.38	1.46
11	120	5	4.2	7.80 ± 3.97	8.68 ± 2.63	3.34 ± 4.99	30.3	B	0.26	0.35	1.32
12	120	15	4.2	11.08 ± 4.10	7.59 ± 4.25	2.98 ± 4.35	42.5	G	0.27	0.37	1.39
13a	120	10	3.6	15.54 ± 4.74	7.14 ± 2.73	3.03 ± 4.24	38.5	G	0.26	0.36	1.39
13b	120	10	3.6	12.40 ± 3.97	6.99 ± 2.74	2.97 ± 4.26	38.8	G	0.28	0.39	1.42
13c	120	10	3.6	12.09 ± 4.08	6.93 ± 2.79	3.18 ± 3.29	40.2	G	0.27	0.37	1.40

**Table 3:** MLR models for selected variables: estimated regression coefficients (b) with *p*-values, standardized regression coefficients (β) and the basic model characteristics.

Regression Coefficient (Constant)/Model Parameter	$Y_{1}$		$Y_{2}$		$Y_{3}$		$Y_{4}$		$Y_{5}$	
	Coefficient	*p*-Value	Coefficient	*p*-Value	Coefficient	*p*-Value	Coefficient	*p*-Value	Coefficient	*p*-Value
$b_{0}$ $\beta_{0}$	−93.3966 0.0178	0.361 (ns)	30.9155 −0.0015	<0.001 ***	11.3003 0.0000	<0.001 ***	−64.8662 0.0246	0.144 (ns)	−45.0000 0.0000	0.005 **
$b_{1}\;\left(X_{1}\right)\;\beta_{1}\;\left(X_{1}\right)$	−4.2188 −8.6415	<0.001 ***	−0.2635 −6.2616	<0.001 ***	−0.0725 −0.3614	0.001 **	1.6284 7.6038	<0.001 ***	0.2188 −0.1157	0.011 *
$b_{2}\;\left(X_{2}\right)\;\beta_{2}\;\left(X_{2}\right)$	1.3080 −0.6524	0.653 (ns)	−0.1182 −1.8881	0.491 (ns)	0.0149 −2.7110	0.885 (ns)	−1.8425 1.5110	0.118 (ns)	−0.4750 1.9670	0.169 (ns)
$b_{3}\;\left(X_{3}\right)\;\beta_{3}\;\left(X_{3}\right)$	185.4160 6.2569	<0.001 ***	−5.3053 −4.8503	0.023 *	−1.5371 −0.3748	0.043 *	10.3711 3.8965	0.485 (ns)	20.4167 8.5640	0.004 **
$b_{11}\;\left(X_{1}^{2}\right)\;\beta_{11}\;\left(X_{1}^{2}\right)$	0.0271 9.1212	<0.001 ***	0.0012 6.8800	<0.001 ***	–	(ns)	−0.0083 −8.0185	<0.001 ***	–	(ns)
$b_{22}\;\left(X_{2}^{2}\right)\;\beta_{22}\;\left(X_{2}^{2}\right)$	0.1386 0.9664	0.038 *	0.0134 1.6753	0.002 **	0.0145 2.7490	<0.001 ***	−0.0566 −1.1837	0.037 *	−0.0200 −1.8780	0.057 (ns)
$b_{33}\;\left(X_{3}^{2}\right)\;\beta_{33}\;\left(X_{3}^{2}\right)$	−20.6202 −6.3651	<0.001 ***	0.9863 5.1313	<0.001 ***	–	(ns)	−4.4550 −3.8149	0.019 *	−2.0833 −8.3460	0.011 *
$b_{12}\;(X_{1}X_{2})\;\beta_{12}\;(X_{1}X_{2})$	−0.0520 −0.2600	0.004 **	0.0015 0.1380	0.101 (ns)	–	(ns)	−0.0077 −0.1199	0.192 (ns)	–	(ns)
$b_{23}\;(X_{2}X_{3})\;\beta_{23}\;(X_{2}X_{3})$	0.8419 0.1291	0.115 (ns)	−0.1025 −0.2833	0.002 **	−0.0856 −0.3589	<0.001 ***	1.1762 0.5370	<0.001 ***	0.2500 0.5249	0.009 **
$b_{13}\;\left(X_{1}X_{3}\right)\;\beta_{13}\;\left(X_{1}X_{3}\right)$	−0.4000 −0.2513	0.003 **	−0.0032 −0.0346	0.684 (ns)	0.0174 0.2926	0.005 **	0.0905 0.1613	0.099 (ns)	−0.0625 −0.5249	0.009 **
$R^{2}$	0.813		0.810		0.679		0.775		0.866	
$\mathrm{adjusted}\;R^{2}$	0.761		0.756		0.625		0.708		0.732	
*p*-value	<0.001 ***		<0.001 ***		<0.001 ***		<0.001 ***		0.012 *	

Note: (ns) non-significant, *p* > 0.05; *** *p* < 0.001; ** *p* < 0.01; * *p* < 0.05.

**Table 4:** Box–Behnken experimental design, observed (O) * and predicted (P) responses for each experimental run, along with their percentage prediction errors (PPE).

Run	Pattern of Coded Factors (X_1_, X_2_, X_3_)	Y_1_			Y_2_			Y_3_			Y_4_			Y_5_		
		O (μm)	P (μm)	PPE (%)	O (μm)	P (μm)	PPE (%)	O (μm)	P (μm)	PPE (%)	O (%)	P (%)	PPE (%)	O (Grade)	P (Grade)	PPE (%)
1	−1, −1, 0	9.1	11.4	−20.3	7.67	7.65	0.2	3.60	3.69	−2.3	35.2	34.2	2.7	3.0	2.5	20.0
2	+1, −1, 0	26.3	35.2	−25.1	8.28	8.38	−1.2	3.65	3.30	10.8	32.3	31.5	2.3	2.0	2.3	−11.1
3	−1, +1, 0	39.4	30.6	28.9	7.07	6.96	1.5	3.48	3.65	−4.6	38.4	39.1	−1.9	3.0	2.8	9.1
4	+1, +1, 0	35.8	33.5	6.9	8.27	8.29	−0.2	3.22	3.26	−1.2	32.4	33.4	−2.8	2.0	2.5	−20.0
5	−1, 0, −1	5.6	6.9	−18.8	7.20	7.06	2.0	3.70	3.70	0.1	36.4	37.0	−1.6	1.0	2.6	−61.9
6	+1, 0, −1	32.1	29.8	7.6	8.42	8.16	3.2	2.68	2.89	−7.2	30.0	30.6	−1.9	3.0	2.6	14.3
7	−1, 0, +1	11.1	13.3	−17.0	7.34	7.60	−3.5	3.20	2.92	9.8	36.3	35.9	1.1	3.0	3.4	−11.1
8	+1, 0, +1	18.3	17.0	7.6	8.33	8.55	−2.5	3.02	2.94	2.5	34.8	33.9	2.6	2.0	1.6	23.1
9	0, −1, −1	12.7	9.1	39.6	7.19	7.35	−2.2	3.47	3.42	1.5	37.2	37.6	−0.9	2.0	2.1	−5.9
10	0, +1, −1	8.2	12.8	−35.8	7.33	7.58	−3.2	4.14	3.90	6.3	35.2	33.9	3.9	1.0	0.9	14.3
11	0, −1, +1	7.8	0.9	748.9	8.68	8.43	2.9	3.34	3.57	−6.5	30.3	31.6	−4.2	1.0	1.1	−11.1
12	0, +1, +1	11.1	14.7	−24.6	7.59	7.43	2.2	2.98	3.02	−1.2	42.5	42.0	1.2	3.0	2.9	4.3
13a	0, 0, 0	15.5	13.3	16.5	7.14	7.01	1.9	3.03	3.11	−2.5	38.5	39.3	−2.1	3.0	3.0	0.0
13b	0, 0, 0	12.4	13.3	−7.1	6.99	7.01	−0.3	2.97	3.11	−4.6	38.7	39.3	−1.4	3.0	3.0	0.0
13c	0, 0, 0	12.1	13.3	−9.4	6.93	7.01	−1.1	3.18	3.11	2.2	40.2	39.3	2.3	3.0	3.0	0.0
Goal		>10 μm; Minimize			Minimize			Minimize			Minimize			Maximize		

* Mean values after exclusion of the outliers.

## Data Availability

Data are contained within the article or Supplementary Materials.

## Supplementary Materials

The following supporting information can be downloaded at: https://www.mdpi.com/article/10.3390/pharmaceutics15020496/s1, Table S1: Results of experiment for variables measured in 3 replications: raw data with indicated outliers.
