# Supplementary material for: Optimization of Spray Drying Process Parameters for the Preparation of Inhalable Mannitol-Based Microparticles Using a Box-Behnken Experimental Design
Source: Pharmaceutics. 2023 Feb 2;15(2):496. doi: 10.3390/pharmaceutics15020496 (PMC9960250; doi:10.3390/pharmaceutics15020496)
Supplement: Supplementary file 1 [file pharmaceutics-15-00496-s001.zip › pharmaceutics-2139030-supplementary.pdf]

Table S1. Results of experiment for variables measured in 3 replications: raw data with indicated outliers

Run	**Drying temperature (X_1)** [°C]	**Pump speed (X_2)** [ml/min]	**Air speed (X_3)** [m/s]	**S (Y_1)** [μm]	**MMAD (Y_2)** [μm]	**NMAD (Y_3)** [μm]	**MMAD RSD (Y_4)** [%]
1	100	5	3.6	11.13	7.50	3.40	36.4
1	100	5	3.6	9.16	8.00	3.80	33.3
1	100	5	3.6	7.09	7.50	6.05*	35.8
2	140	5	3.6	22.12	8.67	4.30*	29.9
2	140	5	3.6	15.32*	8.10	3.78	33.1
2	140	5	3.6	30.54	8.06	3.52	33.8
3	100	15	3.6	40.68*	7.23	3.40	38.3
3	100	15	3.6	40.01	6.98	3.53	38.4
3	100	15	3.6	38.77	6.98	3.52	38.4
4	140	15	3.6	38.20	8.06	3.16	34.3
4	140	15	3.6	35.68	8.35	3.20	31.5
4	140	15	3.6	33.52	8.40	3.30	31.5
5	100	10	3	6.26	7.20	3.60	36.4
5	100	10	3	5.22	7.20	3.60	36.2
5	100	10	3	5.33	7.20	3.90	36.6
6	140	10	3	36.01	8.67	2.50	29.5
6	140	10	3	30.67	8.30	2.64	30.6
6	140	10	3	29.53	8.30	2.90	43.1*
7	100	10	4.2	12.74	7.00	3.40	35.8
7	100	10	4.2	10.81	7.23	2.80	37.9
7	100	10	4.2	9.69	7.77	3.40	35.3
8	140	10	4.2	20.71	8.00	3.05	36.1
8	140	10	4.2	15.21	8.67	3.00	33.4
8	140	10	4.2	19.12	7.50*	3.00	37.6*
9	120	5	3	8.47	7.50	3.60	35.5
9	120	5	3	15.78	7.10	3.40	38.4
9	120	5	3	14.00	6.98	3.40	37.7
10	120	15	3	8.69	7.50	4.22	35.0
10	120	15	3	8.74	7.00	4.10	36.2
10	120	15	3	7.23	7.50	4.10	34.3
11	120	5	4.2	9.77	8.98	3.05	29.9
11	120	5	4.2	14.81*	8.67	3.30	31.1
11	120	5	4.2	5.83	8.40	3.65	29.9
12	120	15	4.2	10.89	7.70	2.95	47.5
12	120	15	4.2	11.89	7.77	3.05	81.9*
12	120	15	4.2	10.44	7.30	2.95	37.5
13a	120	10	3.6	16.87	6.98	3.00	39.1
13a	120	10	3.6	14.43	7.30	3.05	37.8
13a	120	10	3.6	15.33	8.00*	3.05	33.6*

13b	120	10	3.6	15.68	7.00	3.10	36.9
13b	120	10	3.6	13.97	8.06*	3.10	34.9*
13b	120	10	3.6	7.55	6.98	2.70	40.5
13c	120	10	3.6	15.49	7.00	3.28	40.4
13c	120	10	3.6	10.27	7.00	3.16	40.0
13c	120	10	3.6	10.53	6.80	3.10	40.1

* Outlier value.
